# 3D rGO and rGO/TiO_2_–Modified Melamine Sponges: Sorbents for Oil and Emerging Organic Pollutant Removal

**DOI:** 10.1021/acsomega.5c11602

**Published:** 2026-01-23

**Authors:** Kelly Leite dos Santos Castro Assis, Thayane Almeida de Medeiros, Druval Santos de Sá, Carolina Carvalho de Mello, Nádia Cristina Da Silva Iack, Adriana Maria da Silva, Renata Antoun Simão, Braulio Soares Archanjo, Carlos Alberto Achete

**Affiliations:** † Materials Division, 119536 National Institute of Metrology, Quality and Technology (Inmetro), Duque de Caxias, Rio de Janeiro 25250-020, Brazil; ‡ Department of Materials Science and Engineering, Federal University of Rio de Janeiro (UFRJ), Rio de Janeiro, Rio de Janeiro 21941-909, Brazil

## Abstract

Environmental disasters resulting from the exploitation of natural resources, such as oil spills, have severe and lasting impacts on ecosystems. In this work, melamine sponges were modified with reduced graphene oxide (rGO) and titanium dioxide nanosheets (TiO_2_) to develop multifunctional materials for environmental remediation. Prior to TiO_2_ modification, the sponges were coated with rGO of different sheet sizes to evaluate the influence of rGO dimensions on surface coverage and material properties. Graphene oxide was reduced using ascorbic acid as a green reducing agent, and the process was accelerated in a microwave reactor. The resulting 3D material exhibited hydrophobic and oleophilic characteristics, enabling efficient and selective oil absorption from water. Structural and morphological characterizations confirmed the successful modification of the sponge, which displayed an outstanding oil uptake capacity of approximately 85 times its own weight. In addition to oil sorption, the material demonstrated high adsorption efficiencies for various organic contaminants, including methylene blue (95%), rhodamine B (99%), ibuprofen (75%), bisphenol A (71%), and tetracycline (73%). It also exhibited excellent photocatalytic activity toward methylene blue (95%) and maintained remarkable reusability, preserving high removal efficiencies after nine consecutive cycles for RhB (97%), IBP (71%), and TC (70%). These results highlight the potential of rGO/TiO_2_-modified melamine sponges as versatile and sustainable platforms for oil–water separation and the remediation of organic pollutants in aqueous media.

## Graphical abstract

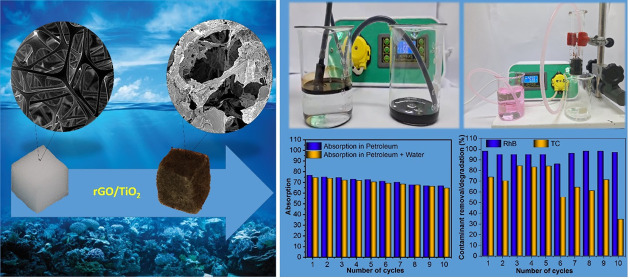

## 1 Introduction

The increasing global demand for oil and its derivatives has led to frequent oil spills and industrial wastewater contamination, resulting in serious environmental and ecological concerns. A critical step in reducing the impact of oil contamination in water is effectively removing oil, which requires the development of advanced materials with selective sorption properties. In this regard, the creation of porous materials with selective sorption properties, particularly hydrophobic and oleophilic characteristics, has attracted significant attention in the recent literature due to their high efficiency in separating oil from water.

Among organic pollutants, oil is a major contributor to water contamination, mainly originating from improper disposal of cooking oil and offshore exploration activities.^1–3^ Oil spills cause severe ecological damage by forming surface layers that hinder light penetration and disrupt photosynthetic processes, leading to eutrophication and significant harm to aquatic organisms.^2^ In addition to environmental impacts, oil spills and chemical leaks pose risks to human health, highlighting the urgent need for efficient and safe remediation strategies.

Nanoscience offers effective solutions for oil spill remediation through the development of advanced materials. Three-dimensional (3D) porous materials are particularly suitable for oil/water separation due to their high porosity, large surface area, and tunable wettability, enabling efficient and selective oil absorption.^4–6^ Polymeric sponges such as polyurethane,^7^ melamine sponges (MS),^8^ poly­(vinyl alcohol) sponges,^9,10^ and polypropylene sponges^11^ have been reported as effective absorbents, highlighting their potential for scalable and sustainable environmental remediation.

Melamine sponges (MS) are commercially available three-dimensional porous materials characterized by superhydrophilicity and underwater superoleophobicity.^12^ When prewetted, MS exhibit high water uptake and selective oil repellence, making them suitable for oil/water separation.^13^ However, direct oil absorption requires surface modification to impart hydrophobic and oleophilic properties. Various modification strategies have been reported, including silane treatments,^14^ graphene oxide (GO),^13^ graphene oxide combined with silver (Ag),^6^ and Fe_3_O_4_/Ag composites.^8^

GO is a versatile precursor for the design of sorbent materials due to its oxygen-rich surface, which contains functional groups such as carboxyl, epoxide, carbonyl, and hydroxyl that promote strong interactions with porous substrates, including polymeric sponges.^15^ These interactions enable effective functionalization of three-dimensional matrices, improving oil–water separation performance. Upon reduction, GO is converted into reduced graphene oxide (rGO), which exhibits enhanced hydrophobicity and electrical conductivity and can be obtained through chemical, electrochemical, or thermal reduction methods.^16^ rGO was selected due to its higher electrical conductivity, increased hydrophobicity, and enhanced π–π interactions compared to GO. These properties are particularly advantageous for oil adsorption and for promoting efficient charge transfer during photocatalytic processes. The reduction of oxygen-containing functional groups also improves chemical stability and favors stronger interactions with nonpolar organic contaminants. Graphene-based porous materials, such as aerogels and hydrogels, have been explored for water purification applications,^17^ supporting the use of rGO-modified 3D structures for environmental remediation. Graphene, one of the earliest investigated two-dimensional (2D) nanomaterials, has attracted attention in water treatment mainly due to its high surface area and favorable electronic properties, which contribute to adsorption and interfacial interactions.^18–21^ In this work, these characteristics are not explored in isolation, but rather leveraged within a three-dimensional porous architecture, where graphene-based materials act as functional modifiers to enhance the performance of the 3D sorbent matrix for environmental applications.

The modification of porous matrices with metal oxides is an effective strategy to enhance the performance of sorbent materials for oil/water separation and the removal of emerging organic pollutants.^22–25^ Among them, titanium dioxide (TiO_2_) is particularly attractive due to its chemical stability, availability, hydrophilicity, and photocatalytic activity. In this study, TiO_2_ nanosheets were incorporated into rGO-modified melamine sponges to integrate complementary functionalities: rGO provides oleophilicity and electrical conductivity, enabling efficient oil absorption, while TiO_2_ introduces hydrophilicity and photocatalytic capability, allowing the degradation of adsorbed organic contaminants. This combination results in a multifunctional 3D material suitable for oil removal and pollutant remediation in aqueous media. Based on this design concept, melamine sponges were modified using graphene oxide (GO) as a precursor to ensure uniform coating of the porous skeleton in aqueous media, followed by reduction to rGO to impart oleophilic properties. Microwave-assisted processing enabled rapid and efficient fabrication of the sponges. The subsequent incorporation of TiO_2_ conferred photocatalytic functionality, enabling UV-driven degradation of contaminants and facilitating material regeneration and reuse. The performance of the sponges was evaluated for petroleum and oil absorption, as well as for the removal of emerging organic contaminants, including methylene blue (MB), rhodamine B (RhB), ibuprofen (IBP), bisphenol A (BPA), and tetracycline (TC).

## 2 Materials and Methods

### 2.1 Synthesis of Graphite Oxide

Graphite oxide was synthesized following Hummers’ method.^26^ Initially, 18.4 mL of concentrated sulfuric acid (H_2_SO_4_, 95–98%, Sigma-Aldrich) was added to a mixture of graphite (0.4 g) and sodium nitrate (NaNO_3_, 99%, Neon) in an ice bath. Potassium permanganate (KMnO_4_, 99%, Sigma-Aldrich, 1.2 g) was then slowly introduced while maintaining the reaction temperature below 20 °C. The mixture was subsequently warmed to 35 °C and stirred for 30 min. Water (18.2 mL) was gradually added, triggering an intense exothermic, increasing the reaction temperature to 98 °C. External heating was applied to sustain this temperature for 15 min. To terminate the reaction, the mixture was transferred to an ice bath for 10 min and quenched by adding a hydrogen peroxide solution (55.3 mL of water and 0.4 mL of 30% H_2_O_2_). The resulting product was filtered through metallic sieves with mesh openings of 500 μm, 300 μm, 250 μm, and 106 μm. The solid was then washed with an HCl solution to remove impurities, followed by repeated washing with water and centrifugation until the supernatant reached pH ∼6. Finally, the material was frozen and lyophilized to obtain a dry product.

### 2.2 TiO_2_ Nanosheets Synthesis

Titanium dioxide was synthesized using the graphene oxide in-plane confined growth method.^27^ Initially, 120 mg of graphene oxide was dispersed in 240 mL of ethanol and stirred for 1 h. Subsequently, 250 μL of titanium­(IV) butoxide was added, and the mixture was stirred for 24 h. After this step, the suspension was washed with ethanol and water to remove weakly bound precursor molecules from the GO surface, leaving the more strongly adhered species intact. The material was then lyophilized. The resulting powder was then calcined in air at 500 °C (2 h). During calcination, the GO was removed by combustion, leading to the formation of titanium oxide nanosheets.

### 2.3 Preparation of GO Dispersions

GO dispersions were prepared by exfoliating graphite oxide (1 mg mL^–1^) in Milli-Q ultrapure water using an ultrasonic bath (Limp Sonic Cleaner, Model LS-3DA-1/X, São Paulo, Brazil) operating at 70 W. Sonication times of 2, 4, and 6 h were employed to evaluate their effect on the absorption properties of the resulting sponges. Previous results from our group demonstrated that longer sonication times lead to smaller GO sheets.^28^

GO/TiO_2_ dispersions were prepared using a similar procedure. Briefly, 30 mg of GO was dispersed in water (1 mg mL^–1^) and sonicated for 2 h. Subsequently, 1 mL of a TiO_2_ solution (1 mg mL^–1^ in water) was added, and the mixture was further sonicated for 2 h.

### 2.4 Melamine Sponge Modification

For this step, the following materials were used: Clink Max Clean MS, Sigma-Aldrich 99% ascorbic acid as the reducing agent, and an Anton Paar Monowave 300 microwave reactor. Figure 1 illustrates the overall process of modifying the melamine sponge to obtain a hydrophobic material. The sponge was modified using two different solutions: a GO dispersion and a GO + TiO_2_ dispersion. The preparation steps were identical for both solutions.

**Figure 1:**
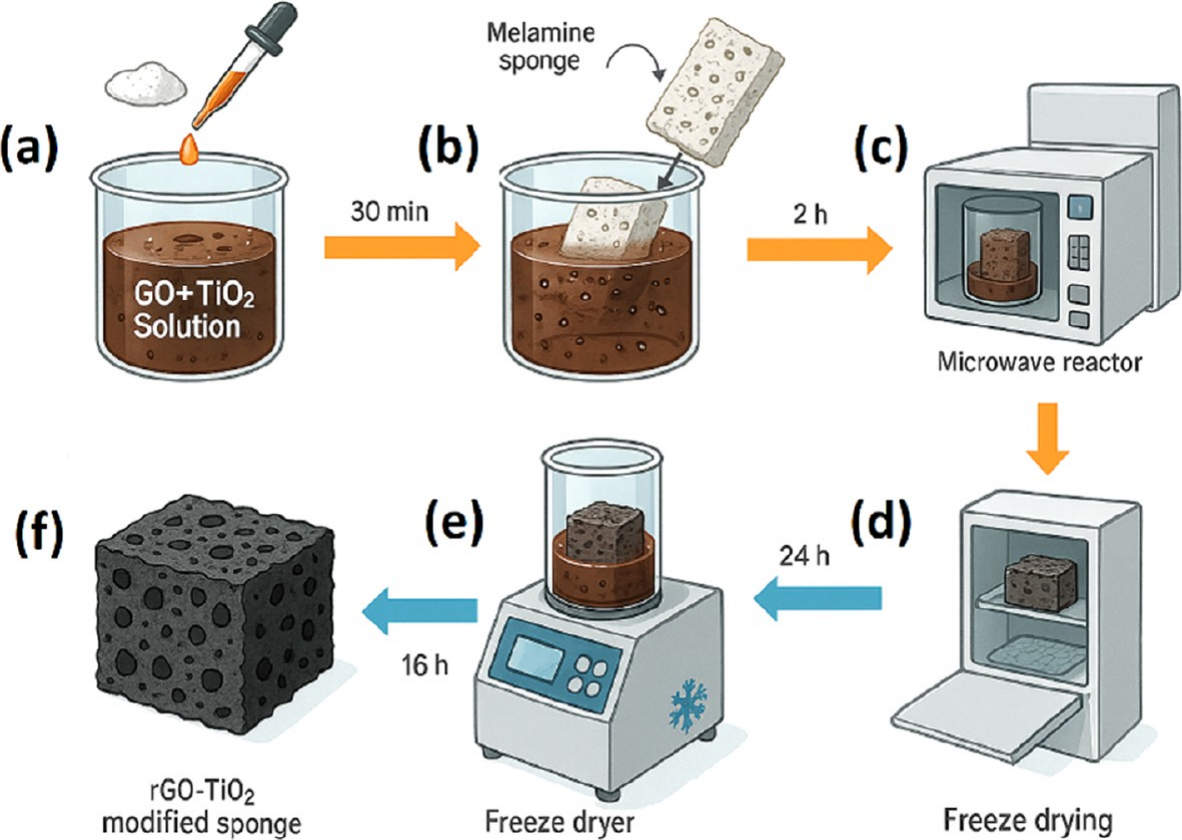
Schematic representation for production of a melamine sponge modified with rGO and rGO/TiO_2_. (a) LAA is added to the dispersion; (b) Melamine sponge is placed in a; (c) reaction occurs in a microwave reactor in order to provide GO reduction into sponge; (d) sponge after GO reduction; (e) drying of the sponges in the freeze-dryer; (f) modified melamine sponge ready for use.

The preparation of the modified sponge begins with the tranfer of 5 mL of the selected solution into a glass vial. Subsequently, 50 mg of l-ascorbic acid (LAA) is added (Figure 1a). LAA serves as a reducing agent, with elevated temperature in a microwave reactor, it facilitates the effective reduction of GO incorporated into the melamine structure.^29,30^

The mixture of GO and LAA is left undisturbed for 30 min in a sealed vial to ensure complete dissolution of LAA in the GO solution. After this period, a 1 cm^3^ melamine sponge (Figure 1b) is immersed in the mixture. The vial is sealed with a silicone septum and allowed to stand for 2 h, enabling full absorption of the solution by the sponge.

Next, the vial containing the mixture is placed in the MW reactor (Figure 1c), programmed with three stages. In the first stage, the temperature of the solution is gradually increased, reaching 110 °C within 5 min. In the second stage, the solution is agitated while maintaining a temperature of 110 °C for 25 min. Finally, in the last stage, the sponge is cooled down. The entire process in the microwave reactor lasts for 40 min. During the temperature increase, the thermochemical reduction of GO occurs.

After the process in the MW reactor, the sponge is placed in a beaker with 50 mL of ultrapure water at 500 rpm for 5 min; this process is repeated five times. Subsequently, the sponge is transferred to a falcon tube and placed in the freezer (Figure 1d). It undergoes a freeze-dryer for 16 h or until all the liquid is removed (Figure 1e). Upon completion of the drying process, the sponges are ready for use (Figure 1f).

### 2.5 Characterization

The electron microscopy characterization of the sponges was performed at the Microscopy Laboratory Center of the Materials Division at INMETRO. Scanning electron microscopy (SEM) analyses were conducted using a Zeiss HIM Orion NanoFab helium ion microscope, equipped with a secondary electron detector and a charge compensation system. The microscope operated at a current of 50 pA and an accelerating voltage of 30 kV. SEM characterization of TiO_2_ and GO nanosheets was carried out using a FEI Magellan 400 L, operated at 98 pA and 5 kV.

X-ray diffraction (XRD) analyses were performed on a Bruker D8-Focus diffractometer using Ni-filtered Cu–Kα radiation (λ = 1.5406 Å), with a step size of 0.02° and a 2θ scanning range from 5° to 40°. Thin films were prepared by drop-casting the aqueous suspensions onto Si wafers.

Raman spectroscopy was conducted with a Witec spectrometer using a 514.5 nm laser in a backscattering configuration and a microscope equipped with a 20× objective. To avoid local heating and sample damage, the laser power was maintained below 0.5 mW. Spectra were acquired with a 10 s integration time and 10 accumulations, covering the range from 100 to 3600 cm^–1^.

Fourier-transform infrared (FT-IR) spectroscopy was performed using a Bruker Alpha II spectrometer. Measurements were taken in the mid-infrared region from 400 to 4000 cm^–1^, with 31 scans per minute, using attenuated total reflectance (ATR).

X-ray photoelectron spectroscopy (XPS) analyses were carried out under ultrahigh vacuum using an Omicron Nano-Technology system with an Al Kα X-ray source (1486.6 eV) operated at 300 eV. Data processing was performed using CasaXPS software.

Water contact angle measurements were performed using an NRL A-100-00 goniometer (Ramé-Hart) to evaluate the hydrophobicity of the sponges.

### 2.6 Absorption Test with Soybean Oil, Palm Oil and Petroleum

The tests were conducted using palm oil to evaluate the absorption capacity of sponges prepared with GO-2h, GO-4h, and GO-6h. Initially, the absorption capacity of the unmodified sponge in water was assessed. Equation 1 was then used to calculate absorption, allowing comparison of the hydrophobicity levels between the modified and unmodified sponges.

1 $$A\left(w/w\right)=\frac{{\mathrm{W̲}}_{f}-{\mathrm{W̲}}_{i}}{{\mathrm{W̲}}_{i}}$$

${\mathrm{W̲}}_{i}$ = initial mass; ${\mathrm{W̲}}_{f}$ = final mass

The absorption capacity of the modified sponges was evaluated in a mixture of palm oil and water, in pure palm oil, and in pure water for a duration of 10 min (Figure 2a). This test was carried out to assess the oil absorption performance of the sponges and to compare their water absorption with that of the unmodified sponge.

**Figure 2:**
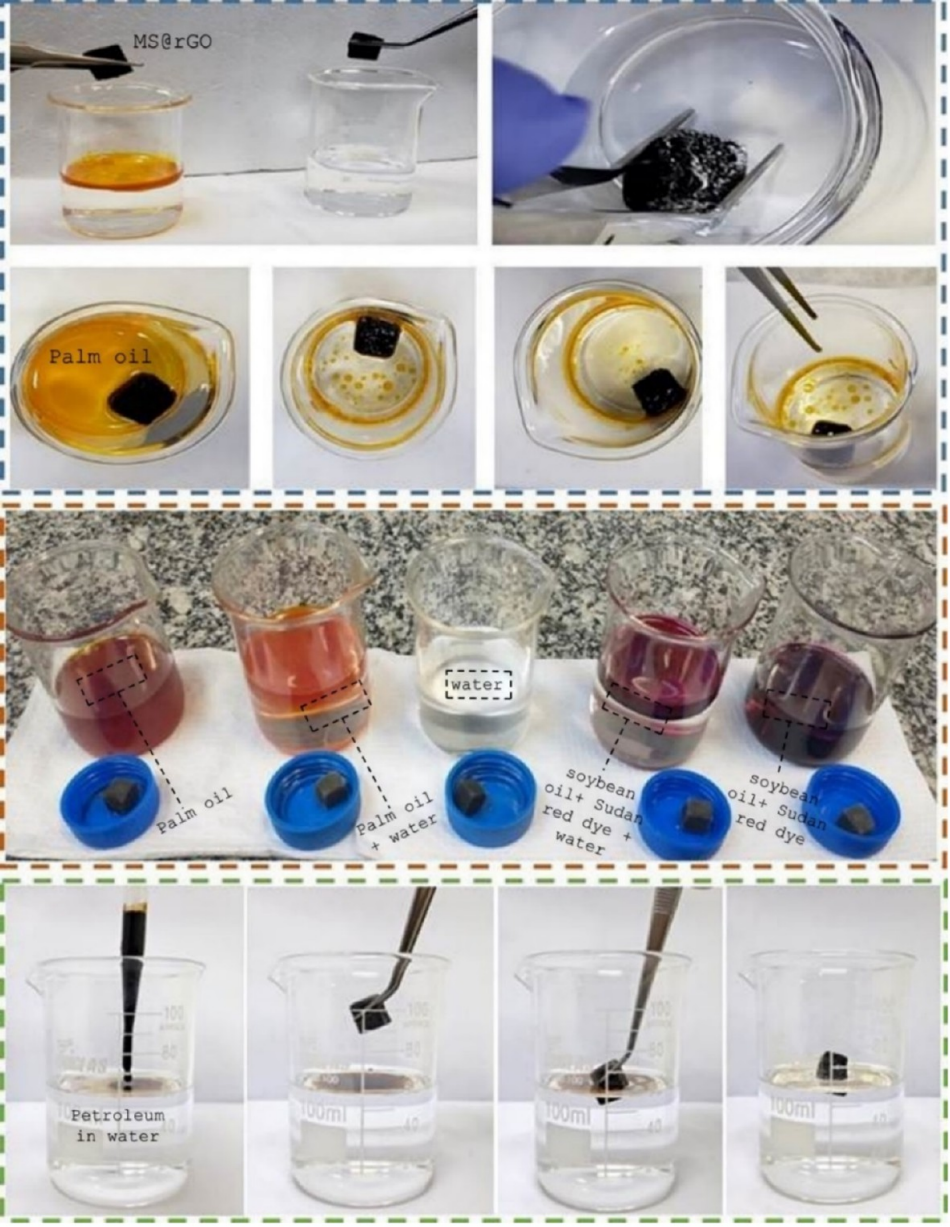
(a) Absorption test of sponges produced with GO exfoliated for 4 h in palm oil (blue dotted region); (b) Absorption test of sponges produced with GO exfoliated for 4 h from left to right: palm oil, palm oil and water, water, soybean oil dyed with Sudan red/water and soybean oil dyed with Sudan red (orange dotted region); (c) Petroleum absorption test (green dotted region).

The same immersion procedure was performed using soybean oil (Figure 2b), which was dyed with Sudan Red 7B to improve visualization during the experiment. Subsequently, the sponges were tested with petroleum (Figure 2c). Absorption tests were carried out following the same procedure as for soybean oil and palm oil.

Flow tests were performed using the OFA 100D flow simulator, with an output set at 0.001 and a flow rate of 27%.

### 2.7 Removal Test of Organic Contaminants by Adsorption/Photocatalysis under Continuous Flow Conditions

To evaluate the combined efficiency of the adsorption and photocatalytic activity processes of the MS-rGO-4h-TiO_2_-NS material in the removal of emerging organic contaminants in aqueous medium, a continuous flow system was developed, consisting of: a 50 mL glass syringe, MS-rGO-4h-TiO_2_-NS (Ø = 25 mm, *h* = 10 mm), an OFA 100 D peristaltic pump (Interlab, Brazil), 30–80 mL of an organic contaminant solution. The contaminants (IBP, BPA, MB, RhB, and TC) were individually tested at different concentrations under a 9 W UV-B lamp.

The adsorption capacity (qt) of the organic contaminants MB, RhB, IBP, BPA, and TC on MS-rGO-4h-TiO_2_-NS was calculated using eq 2, while the kinetic parameters were simulated according to the pseudo-first-order and pseudo-second-order models, expressed by eqs 3 and 4, respectively.

2 $$q_{t}=\frac{V\left(C_{0}-C_{t}\right)}{m}$$

3 $$q_{t}=q_{e,1}\times \left(1-e^{{-k}_{1}t/2,303}\right)$$

4 $$q_{t}=\frac{q_{e,,2k_{2}t}^{2}}{1+k_{2}q_{e,2}t}$$

where *V* (L) represents the volume of contaminant solution; *m* (g) is the weight of MS-rGO-4h-TiO_2_ NS; *C*_0_ (mg L^–1^) and *C*_*t*_ (mg L^–1^) represent the initial concentration and at different time intervals, respectively; *q*_e,1_ and *q*_e,2_ (mg·g^–1^) are the amount of contaminant adsorbed at the corresponding adsorption equilibrium, and *k*_1_ (min^–1^) and *k*_2_ (g·mg^–1^·min^–1^) are the pseudo-first-order and pseudo-second-order rate constants, respectively.

## 3 Results and Discussion

The synthesis of graphite oxide achieved a yield of 58%, enabled by the use of expanded graphite as starting material. This choice of graphite powder over flake graphite is driven by its larger surface area,^31,32^ which enhances interactions with oxidizing agents during GO synthesis, resulting in a higher yield.

The synthesized graphite oxide was characterizated by SEM, XRD, Raman spectroscopy, TEM, and XPS. The corresponding images are presented in Figure 3a–f.

**Figure 3:**
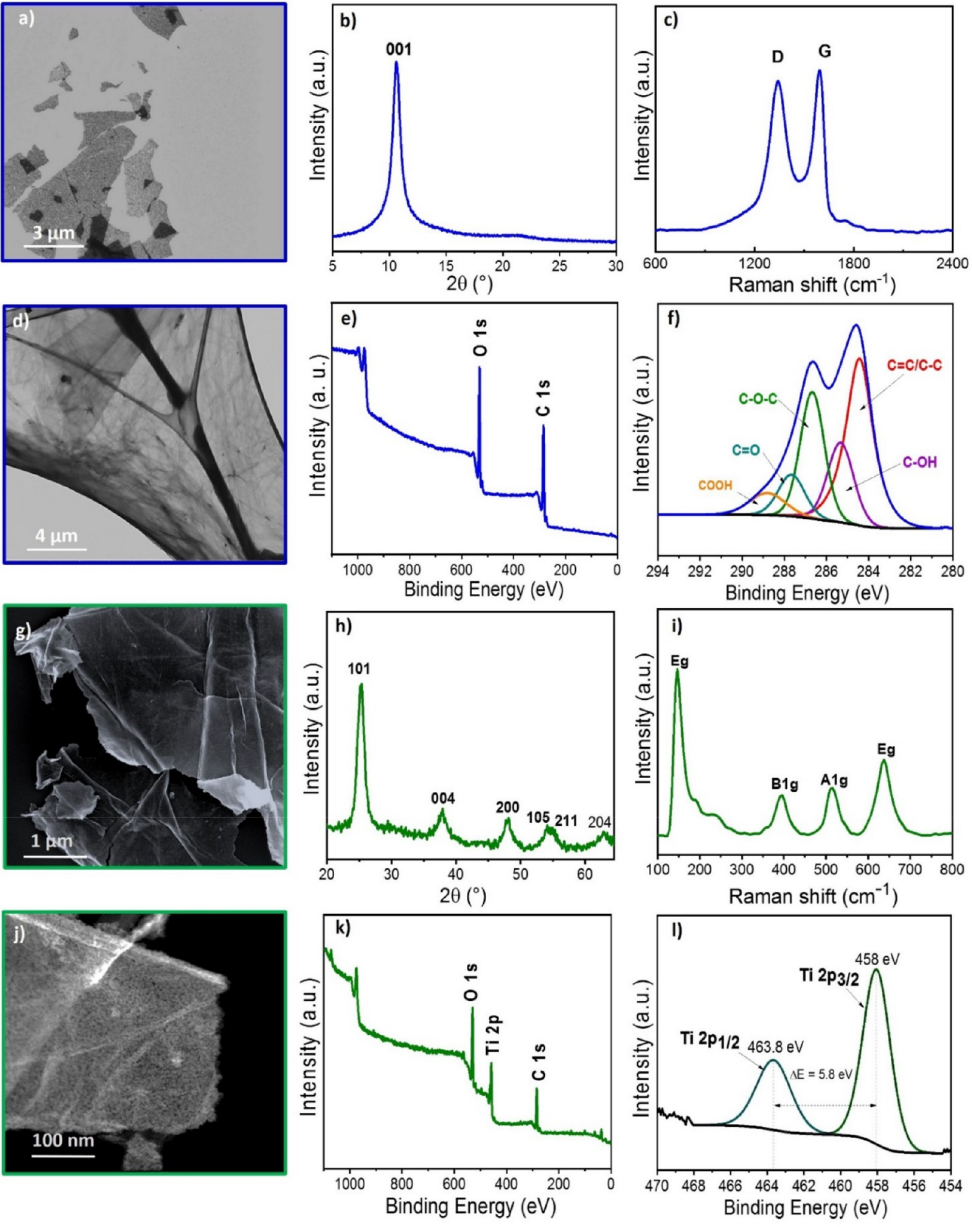
Characterization of graphite oxide: (a) SEM image, (b) XRD, (c) Raman and (d) TEM image, (e) XPS survey spectrum and (f) XPS high resolution C 1s spectrum. Characterization of TiO_2_ nanosheets: (g) SEM image, (h) XRD, (i) Raman analysis, (j) TEM image, (k) XPS survey spectrum and e (l) XPS high resolution Ti 2p spectrum.

The SEM image (Figure 3a) and the TEM image (Figure 3d) demonstrate successfully exfoliation of the graphite oxide through the Hummers-methodology into nanosheets of graphite oxide, forming characteristics layered structures. XRD analysis of graphite oxide (Figure 3b) reveals a diffraction peak at 2θ = 10.36°.^28^ This peak confirms the exfoliation of graphite, which originally exhibits a diffraction peak at 2θ = 26.6°.^16,28,33^ Raman spectroscopy analysis of graphite oxide (Figure 3c) reveals two prominent bands: the D band and the G band. The D band, observed at 1350 cm^–1^, and the G band, centered around 1580 cm^–1^ in structurally perfect materials like graphite, are characteristic of sp^2^-hybridized carbon and reflect the planar structure of graphene.^35^ However, in the graphite oxide spectrum, a blue shift of the G-band to 1603 cm^–1^ is observed, which results from overlap with the D′ band, as the G band remains fixed in position. This shift, combined with the broadening of the G band, indicates the presence of the D′ band, which is associated with stacking faults and/or the presence of heteroatoms. These results provide evidence of structural defects in the carbon lattice, such as the incorporation of oxygen-containing functional groups,^34,35^ as confirmed by XPS analysis. XPS analysis identified the primary oxygen–carbon bonds in the graphite oxide structure, corresponding to oxygen-containing functional groups, including carbonyl, hydroxyl, epoxy, and carboxyl groups.^28,34^

SEM and TEM analyses of TiO_2_ nanosheets (Figure 3g and j, respectively) reveal the formation of highly uniform, well-defined sheets. Additionally, TEM images indicate that the nanosheet surfaces are decorated with nanoparticulate features, likely TiO_2_, as confirmed by XRD analysis identifying the anatase phase of TiO_2_. These morphological characteristics suggest a strong synergy between rGO and TiO_2_, highlighting the effectiveness of the planar-confined growth method for synthesizing metal oxides on GO. The presence of oxygenated moieties on GO facilitates the efficient formation of TiO_2_ nanosheets through strong interactions between these moieties and Ti^2+^ sites. The Ti^2+^ species adsorb onto the oxygenated groups on the GO sheets, promoting controlled nucleation and growth of the nanosheets. Additionaly, after the calcination step, particle nucleation contributes to the growth of TiO_2_ nanosheets.

The XRD pattern of the TiO_2_ nanosheets (Figure 3h) reveals the formation of a crystalline phase, as evidenced by sharp diffraction peaks indicating the presence of larger crystallites. Nevertheless, the broader base of the peaks suggests the coexistence of an amorphous phase and smaller crystallites. Regarding the structural phase, the diffraction peaks at 2θ values of 25.4°, 37.9°, 48.0°, 54.3°, 56.0°, and 62.8° are attributed to the anatase phase of tetragonal TiO_2_. Thus, the sample is predominantly composed of anatase, although the presence of rutile can not be entirely ruled out due to potential overlap or masking of its main diffraction peak at 2θ ≈ 27.4° by the amorphous component.^36^

Raman spectroscopy of the material (Figure 3i) shows peaks characteristic of TiO_2_. Peaks observed at 140 cm^–1^, 430 cm^–1^, 510 cm^–1^, and 633 cm^–1^ correspond to the anatase phase (modes Eg, B1g, B1g, and Eg, respectively). Notably, the bands at 510 cm^–1^, and 633 cm^–1^ are broad and a potential overlapping with Eg and A1g modes of the rutile phase at 445 cm^–1^ and 606 cm^–1^ might occurs.^36^ The process produces nanosheets characteristic of TiO_2_ after high-temperature calcination, which also results in the degradation of graphene.^27^ The calcined material was subsequently used to modify the melamine sponge, as shown in Figure 1. Similarly to XRD, the broad bands indicates a the presence of structural disorder and/or oxygen vacancies in the TiO_2_ structure.

In the first step, the recovery of the melamine sponge was evaluated using modifications with GO exfoliated for different treatment times. Our preliminaries studies demonstrated that the size of GO sheets can influence both electrochemical performance and biological properties.^28,37^ Figure S1 shows the lateral size distribution of GO nanosheets obtained after different reaction times (2, 4, and 6 h). All distributions exhibit a right-skewed profile, typical of particulate systems with wide dimensional variation. The GO 6 h sample presented the highest number of analyzed sheets (N = 3389) and an average size of 41.2 nm, with 93.3% of the sheets smaller than 60,000 nm, indicating the formation of thinner and more uniform sheets after a longer treatment time. For the GO 4 h sample, the average size increased to 82.9 nm, and the distribution was broader, suggesting a lower degree of exfoliation and the presence of larger sheets. The GO 2 h sample, although also showing an average of 41.2 nm, exhibited high dispersion and a smaller number of analyzed sheets. These results demonstrate that the exfoliation time significantly influences the fragmentation and dispersion of GO nanosheets, with the 6 h treatment being the most effective for producing smaller and more homogeneous sheets. Concerning to sponge recovery, the sheet size could impact the extent of modification, and the absorption performance of the sponges was also assessed. The produced sponges were were characterized to evaluate their structural, morphological, and chemical changes (Figure 4).

**Figure 4:**
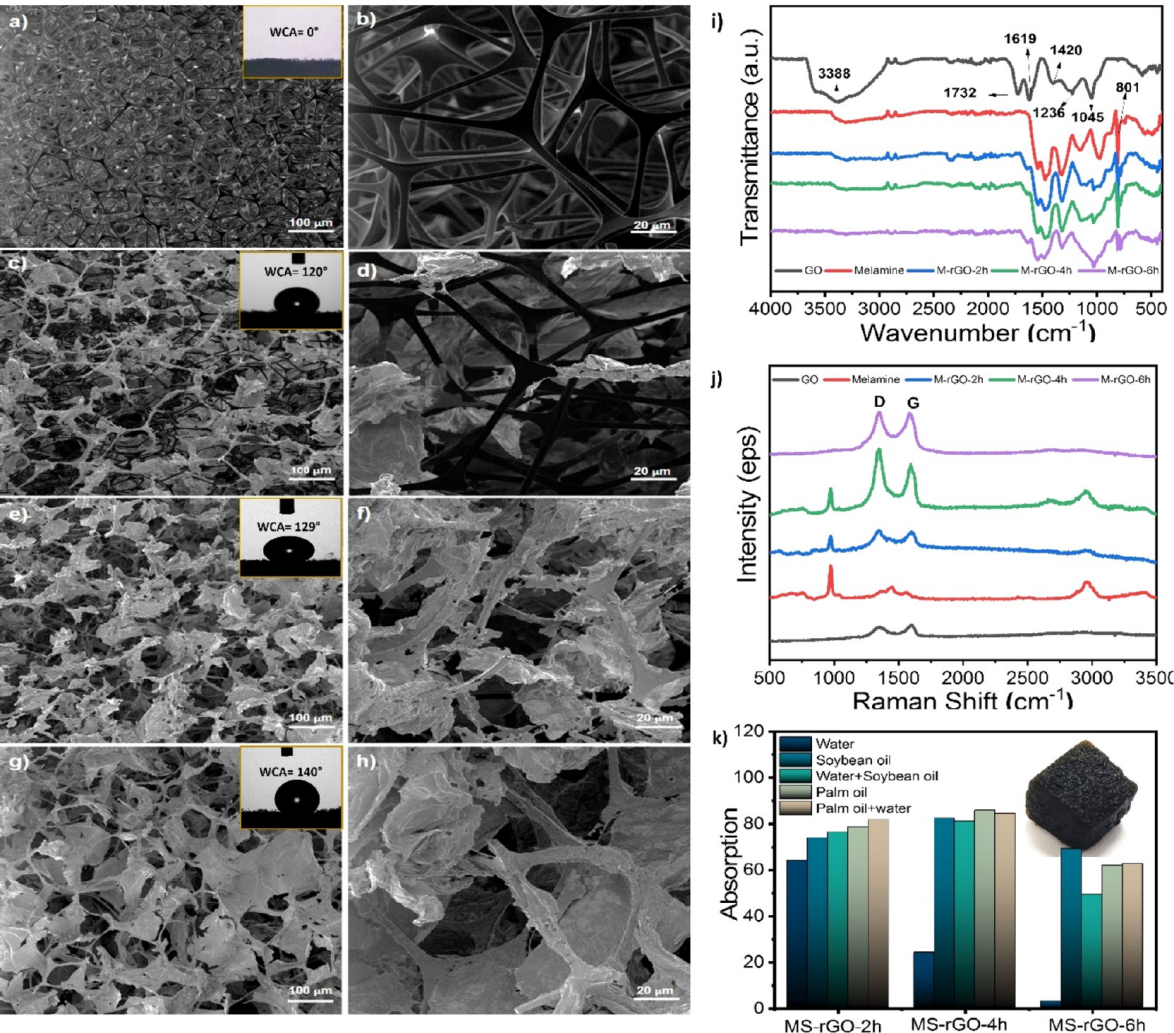
Electron Microscopy images of sponges: (a,b) unmodified; (c,d) modified with graphene oxide exfoliated solution for 2 h; (e,f) modified with graphene oxide exfoliated solution for 4 h; (g,h) modified with graphene oxide exfoliated solution for 6 h; FT-IR (i) and Raman (j) analises of all produced sponges; absorption test of MS-rGO-4h (k).

MEV Imagens of the unmodified sponge (Figure 4a,b), indicates the melamine fibers are clearly defined. In contrast, the sponge modified with rGO-2h (Figure 4c,d) shows only partial coverage of the fibers, with extended areas uncoated. This results in a sponge exhibiting both hydrophobic regions (covered with rGO) and hydrophilic regions (uncoated). Upon time increasing, the sponges modified with GO-4h (Figure 4e,f) and GO-6h (Figure 4g,h), the coverage of the melamine fibers is more efficient compared to GO-2h.

It worth noting that an ultrasonic-microwave synergistic approach for producing rGO-modified melamine sponges has already been reported using ethylenediamine as the reducing agent.^38^ In our work, we employed ascorbic acid (vitamin C) as a greener and more sustaintable reducing agent, highlighting its key role in achieving efficient reductiona and functioanalization of graphene oxide.^34,39^ While ethylenediamine can achieve a higher restoration of the sp^2^-conjugated network, providing higther conductivity, ascorbic acid offers a balance between conductivity and stability, making it ideal for applications where surface modification of rGO is essential. The restoration of the sp^2^-conjugated network with ascorbic acid can be further enhanced by applying heat. Moreover, this contribution establishes an efficient methodology to modify melamine sponges using a green reducing agent in a microwave reactor, combining sustainability with effective material functionalization. The sponge modified with GO-6h (Figure 4g,h) exhibits more extensive fiber coverage, resulting in narrower pore openings within the sponge structure.

The infrared spectra of the samples are shown in Figure 4i. Graphite oxide typically displays a broad absorption band in the range of 3000–3700 cm^–1^, attributed to O–H stretching vibrations, and a band at 1619 cm^–1^, corresponding to H–O–H bending, characteristic of water molecules.^40^ The C=O stretching band between 1700–1750 cm^–1^ is characteristic of carbonyl functional groups, such as aldehydes, ketones, and carboxylic acids. Additionally, C–O bonds are observed at approximately 1230–1215 cm^–1^, 1120–1110 cm^–1^ (epoxy groups), 1414 cm^–1^, 1160 cm^–1^ (carbonyl or carboxyl groups), and 1080–1040 cm^–1^ (alkoxy or epoxy groups), indicating that the original extended π-conjugated system of natural graphite has been disrupted, and oxygen-containing groups have been incorporated into the carbon framework. The C–C aryl stretching vibration may appear at 1420 cm^–1^. These features suggest that graphene oxide is highly hydrophilic and readily absorbs moisture from the surrounding atmosphere, which has important implications for applications in aqueous systems or processes involving water interactions.^41^

In the FT-IR spectra of melamine, absorption peaks at 3469, 3419, 3334, and 3129 cm^–1^ are assigned to –NH_2_ stretching vibrations, while the more intense peaks between 1654 and 813 cm^–1^ correspond to the triazine ring.^42^ After modification, a decrease in these peak intensities is observed. The melamine sponge modified with GO-6h shows lower-intensity peaks, confirming effective coating and corroborating the results observed in microscopy analysis.

Raman spectroscopy enables the selective study of regions predominantly composed of rGO or melamine. The Raman spectra of the analyzed samples exhibit characteristic bands commonly associated with graphene-based materials (Figure 4j). The D-band, observed around 1350 cm^–1^, indicates the presence of lattice defects or disorder, which may arise from vacancies, oxygen-containing functional groups, or adsorbed molecules on the surface.^35,43^ Analogously to the Raman spectrum of graphite oxide, it is observed a blue shift of the G-band to 1600 cm^–1^, characterizing the overlapping with the D′-band stemming from the defective structure and heteroatoms presence such as –NH_2_ groups as demonstrated by FTIR. Peaks at 985 cm^–1^ and 1450 cm^–1^ correspond to the CNC and NCN bending vibrations of the triazine ring in the melamine structure.^40^ As the sponge coating becomes more uniform and efficient, the characteristic peaks of the melamine structure diminish, consistent with observations from FT-IR analysis.

Contact angle analysis is used to evaluate the hydrophobicity of the sponge. A 2.5 mL drop of water was placed on the MS, MS-rGO-2h, MS-rGO-4h, and MS-rGO-6h sponges. The results are shown in Figure 4a, c, e, and g, respectively. For the unmodified melamine sponge, the water droplet was rapidly absorbed, forming an angle of 0° immediately after contact, indicating its strong hydrophilicity.^38^ This corroborates the need for the modification process, since the tests presented in this article require a material that remains afloat and interacts preferentially with hydrophobic compounds. After modification with rGO, the water droplets formed contact angles greater than 90°, confirming the resistance of the sponges to absorb water and evidencing their hydrophobic character, in agreement with previous reports in the literature.^38,44^ These results highlight the efficiency of the modification process, yielding sponges that, when applied in oil spill and oil contamination simulations, exhibit the highest performance, as shown in Figure 2.

In Figure 4k, the absorption capacity of melamine sponges modified with reduced graphene oxide (rGO) at different exfoliation treatment times is presented, with the corresponding SEM micrographs (4c–h), illustrating the surface morphology and structural changes induced by the exfoliation process. The MS-rGO-2h sponge exhibited simultaneous hydrophobic and hydrophilic behavior, absorbing oil while also retaining water, which makes it unsuitable for oil separation applications. It is important to note that this result contrasts with the contact angle shown in Figure 4c, which indicated a considerable degree of hydrophobicity but not hydrophilicity. A possible explanation is that the modification was more effective on the sponge surface than within its internal structure.

The MS-rGO-6h sponge showed strong resistance to water absorption, confirming the hydrophobic character indicated by its contact angle. However, despite this favorable performance in terms of hydrophobicity, the micrographs revealed a significant narrowing of the pores after modification, which hindered the absorption of the tested oils due to the size of their molecules.

Therefore, the sponge that demonstrated the best overall performance was MS-rGO-4h, characterized by strong resistance to water absorption and excellent absorption capacity for different oils, as shown in Figure 4k. For this reason, MS-rGO-4h was selected for the petroleum absorption and photocatalysis tests. The MS-rGO-4h modification with TiO_2_ was investigated at this condition, MS-rGO-4h.

Figure 5 presents the petroleum absorption performance of melamine sponges modified with rGO and rGO + TiO_2_. The flow system used for petroleum absorption tests is illustrated in Figure 5a, while the SEM image of the rGO + TiO_2_-modified sponge is shown in Figure 5b. As observed in Figure 5c,d, the rGO-modified sponge exhibits a higher initial absorption capacity than the rGO + TiO_2_-modified sponge. This difference can be attributed to the partial occupation of the sponge pores by TiO_2_ nanosheets, which reduces pore volume and limits sponge–oil interfacial contact. The adsorption mechanism is governed primarily by hydrophobic–oleophilic interactions between the rGO-modified sponge surface and petroleum components, complemented by π–π interactions between aromatic hydrocarbons and the graphene domains, as well as pore-filling effects within the three-dimensional structure. In the rGO + TiO_2_ composite, although the presence of TiO_2_ slightly decreases the initial oil uptake, it contributes to enhanced structural integrity and surface stability during repeated adsorption–desorption cycles. A gradual decrease in absorption capacity observed over multiple cycles can be attributed to partial pore blockage by residual oil, surface fouling, and the progressive loss of accessible active sites during repeated use. Nevertheless, the incorporation of TiO_2_ nanosheets improves recyclability by mitigating irreversible fouling and facilitating material regeneration. The sponges were regenerated using toluene, a byproduct of petroleum processing, which effectively removes adsorbed oil and enables both sponge reuse and recovery of petroleum for potential industrial reutilization.

**Figure 5:**
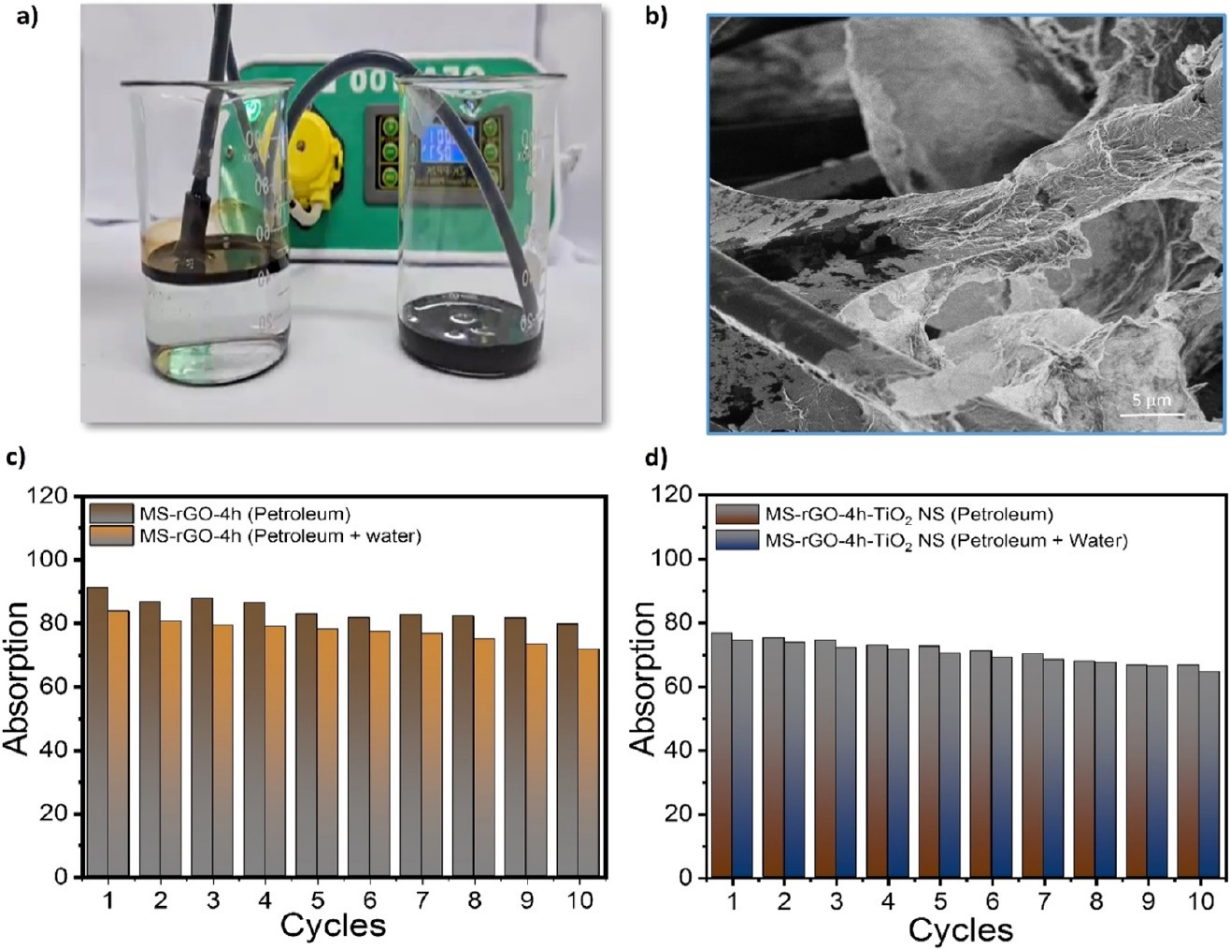
(a) Schematic representation of the oil–water flux system; (b) SEM image of MS-rGO-4h-TiO_2_ NS; and oil absorption performance of (c) MS-rGO-4h and (d) MS-rGO-4h-TiO_2_ NS in petroleum.

Due to the high demand for techniques that can serve as corrective measures in oil spill scenarios, several authors have developed technologies to mitigate the environmental impacts of offshore accidents involving organic compounds, as summarized in Table 1.

**Table 1:** Comparison of Sponge Absorption in Various Studies

material used	synthesis process	absorbed material	absorption capacity (w/w)	cicles sorption	ref
C/P/W@MF	photothermal-driven	silicone oil	79	8	^46^
FGN/PU sponge	solvothermal technique	crude oil	44	50	^30^
rGO-MS	ultrasonic-microwave assisted synthesis	petroleum	72	20	^51^
thiolated G-coated PU sponge	thiolated graphene immersion	chloroform	90	10	^45^
rGO-MS	dip coating followed by chemical reduction	chloroform	149	20	^47^
MS	immersion in an ethanol solution of octadecyltrichlorosilane	light Petroleum	68	50	^48^
rGO-MS	dip-adsorbing and freeze-dried	CCl_4_	133	10	^49^
MF@P(St-DVB)/PDMS	in-situ loading of nanospheres	chloroform	122	50	^50^
MS/TA/APTES/HTDS	ultrasonic	chloroform	195	20	^52^
rGO-MS	graphene oxide reduction with microwave reactor	soybean oil palm oil petroleum	82–91	20	this work

Sponges modified with PU for adsorbing oil components from water have been reported, highlighting their remarkable absorption capacity.^30,45^ However, when comparing PU sponges with MS foam, the results indicate that the higher porosity of melamine foam provides superior absorption values (w/w) compared to PU. Other authors, as well as the present work, have also reported the efficiency of surface-modified melamine sponges for oil removal from water. Another key advantage of MS over PU sponges is their greater flexibility, which enhances their suitability for applications requiring conformability and compressibility. This structural difference significantly impacts both the modification process and the absorption capacity of the material. Our study employs a softer material, which facilitates better adherence of the modifiers within the pores, resulting in improved absorption performance. The same principle explains the superior performance of our modified melamine compared to the PU foam reported in [Zhang *et al.* (2017)], particularly for oil–water separation. As expected, the crude oil absorption capacities reported in the two referenced PU sponge studies (44 and 47 w/w, respectively) were significantly lower than that obtained in this work (91 w/w).

MS modified with graphene oxide or metal oxides have been investigated, achieving excellent results.^38,46–50^ Photothermal materials that reduce intermolecular forces and enhance the absorption rate of organics, have also been developed [Jia *et al*. (2024) and Qi *et al.* (2023)], demonstrating significant improvements in separation efficiency.^51,52^ Although previous studies reported maximum absorption capacities of 79 w/w for oil silicone and 70 w/w for soybean, both denser organic compounds than petroleum, these values were surpassed by the present work. Our modified melamine sponge achieved a superior absorption capacity of 91 w/w for petroleum, demonstrating enhanced performance.

A synergetic methodology using ultrasonication and microwave treatment was utilized for the modification of a melamine sponge with rGO.^38^ Despite some similarities, their approach presents relevant differences compared to ours, such as the concentration of the modifying solution, the reducing agent employed, and the sequence of modification steps. In their method, the exfoliation of graphite and the microwave-assisted reaction were integrated, promoting GO reduction prior to sponge incorporation. In contrast, in our work, the microwave reactor enables continuous stirring of the solution and uniform modification of all sites within the melamine sponge. The reduction process occurs inside the microwave reaction, with the sponge already immersed and impregnated with the rGO solution, ensuring homogeneous reduction throughout the material. This distinction is crucial to the final results, as our approach proved more efficient in separating crude oil from water. Quantitatively, the sponge reported by Song *et al.* (2016) exhibited an absorption capacity of 72 w/w, whereas our sponge reached 91 w/w.^38^

A rGO modified sponge with the GO reduction was achieved by using a thiourea solution after the sponge modification step.^47^ However, thiourea is classified as potentially carcinogenic, generating residues that may pose risks to society. In addition, the sulfur atoms present in thiourea increase the polarity of the material, which could lead to partial dispersion in water during sponge use for the removal of hydrophobic compounds. Nevertheless, it is worth noting that the sponge achieved remarkable absorption when applied to chloroform (149 w/w). For instance, for diesel, which has a density closer to that of crude oil and was also tested by Dehingia *et al.* (2023), the absorption was around 80 w/w, which is lower than the value obtained in the present work.

Super hydrophobic sponges with contact angles above 150° were developed.^48,50^ As observed in this study with the MS-rGO-6h sponge, as the hydrophobic character rises, the melamine pores decrease, hindering the oil molecules penetration into the sponge. Comparing our results with the maximum absorption of 68 w/w for light crude oil [48], 123 w/w for chloroform Hung *et al.* (2023) confrm that the hydrophobic material presented in the present work is promising for oil–water separation, particularly for long-chain organic compounds.^50^

Table 1 presents a compilation of the literature results on the absorption of organic compounds, some of which report higher absorption capacities of those presented here. Nevertheless, these study stands out due its innovative methodology, which employs a rapid thermochemical route using microwave radiation to achieve GO reduction within the sponge just in 40 min. Moreover, sponges demonstrated superior absorption performance when petroleum is used as the target contaminant. Notable, the incorporation of TiO_2_ in the MS-rGO system was crucial in order to enhance the efficiency (Figure 5), improving the stability during recycling cycle, ensuring sustained absorption across multiple uses. To facilitate a clear and systematic comparison with previously reported sorbent materials, a summary of key literature data is provided in Table S1 (Supporting Information). The table compiles relevant parameters such as synthesis approaches, maximum oil adsorption capacities, and additional functional or catalytic properties, allowing an objective evaluation of the performance of the present material within the current state of the art.

Figure 6 shows the flow system and the results of the adsorption/photocatalysis tests for 55 mg of MS-rGO-4h-TiO_2_ NS with solutions of the contaminants methylene blue (MB), rhodamine B (RhB), ibuprofen (IBP), bisphenol A (BPA), and tetracycline (TC) at a flow rate of 10 mL min^–1^. When 80 mL of MB solution (4.5 × 10^–5^ mol L^–1^) passed through the system, a color change from dark blue to colorless indicating effective contaminatnt removal (Figure 6e). However, beyond this volume, the effluent solution turned light blue, signaling the onset of the active sites saturation. After 100 mL of solution had passed, the MB concentration remained constant, indicating complete saturation of the active sites.

**Figure 6:**
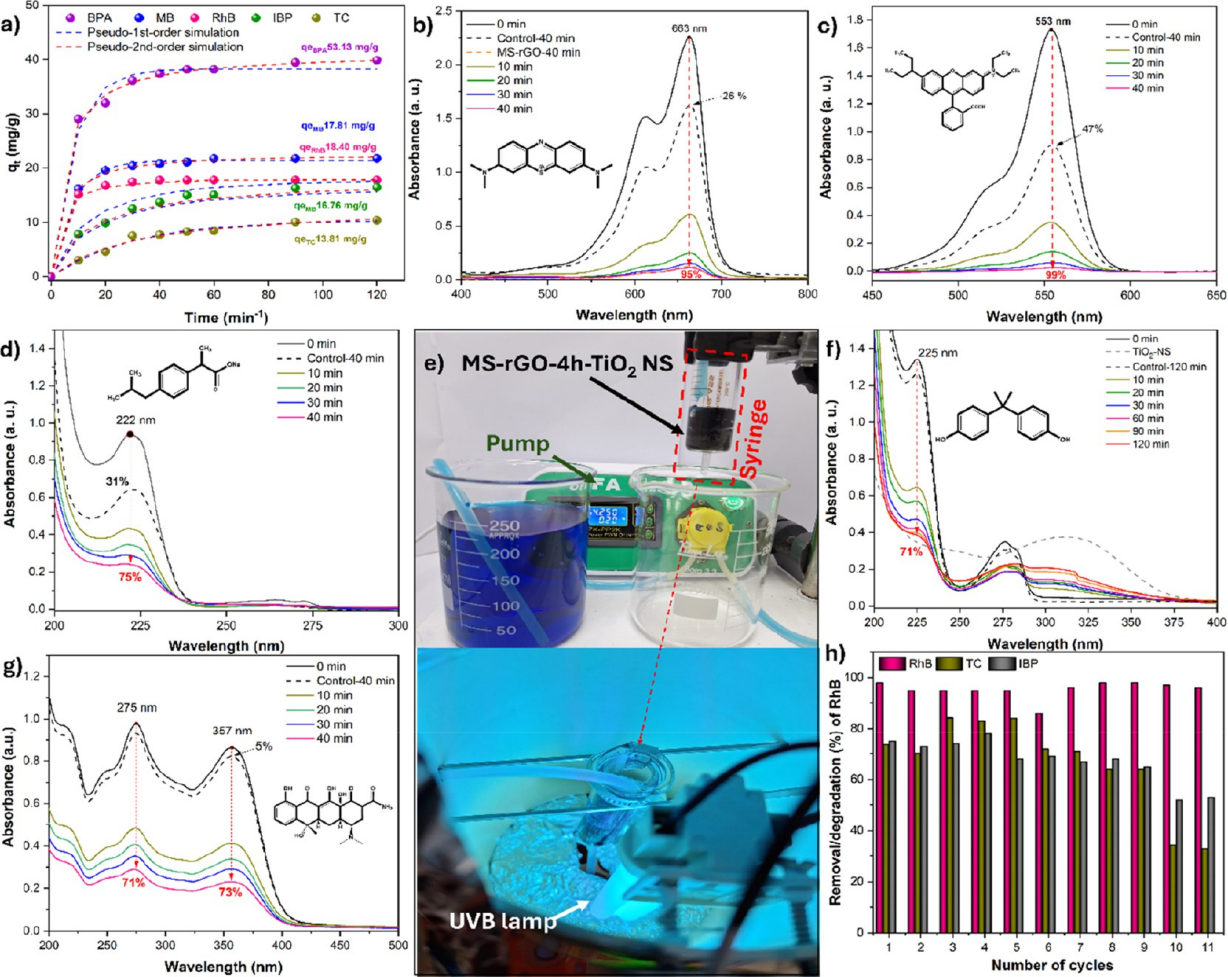
(a–f) UV–vis absorption spectra of MB, RhB, IBP, BPA, and TC at different adsorption time. (g) Adsorption kinetics of MB, RhB, IBP, BPA, and TC onto MS-rGO-4h-TiO_2_ NS in 0 mL of solutions with initial concentrations of 100 mg·L^1^ and 50 mg·L^1^, respectively. (h) Removal efficiency of MB onto Cu-Z-GO-M (1:1) in five cycles.

The adsorption kinetics of MB, RhB, IBP, BPA, and TC on MS-rGO-4h-TiO_2_ NS were investigated, and the results are shown in Figure 6a. The adsorption rates of the organic contaminants increased significantly during the first 10 min and reached adsorption equilibrium after 40 min. The adsorption capacity of the contaminants on MS-rGO-4h-TiO_2_ NS followed the order: BPA (*q*_e_ = 53.13 mg g^–1^) > MB (*q*_e_ = 18.40 mg g^–1^) > RhB (*q*_e_ = 17.81 mg g^–1^) > IBP (*q*_e_ = 16.76 mg g^–1^) > TC (*q*_e_ = 13.81 mg g^–1^). Based on the equilibrium adsorption capacities (*q*_e_) and the pseudo-first-order (*k*_1_) and pseudo-second-order (*k*_2_) rate constants, the adsorption process is best described by the pseudo-second-order model (red line). As expected, the adsorption of these contaminants onto MS-rGO-4h-TiO_2_ NS occurs mainly via chemisorption.^53^ Although the adsorption capacity of MS-rGO-4h-TiO_2_ NS is highly efficient for emerging contaminants, when combined with advanced oxidation processes (AOPs), it further enhances the removal of organic compounds (OCs) from wastewater and facilitates regeneration of active sites saturated by Ocs.^54^ All tests under UVB irradiation in the continuous-flow system were conducted only after the sponge reached complete saturation with a fresh solution of the OCs.

Figure 6a–f show the UV–Vis absorption spectra of MB, RhB, IBP, BPA, and TC at different adsorption times. MS-rGO-4h-TiO_2_ NS exhibited high adsorption capacities for MB (95%), RhB (99%), IBP (75%), BPA (71%), and TC (73%), compared to the controls (unmodified melamine sponge), which reached 26%, 47%, 31%, <1%, and 5%, respectively. The results obtained with TiO_2_ NS alone showed only 20% photocatalytic activity during the tested period, whereas TiO_2_ NS combined with MS-rGO reached 95% for MB. This enhanced performance is attributed to the synergistic effect of combining TiO_2_ NS with rGO, which improves OC photodegradation through enhanced adsorption capacity, charge separation, and efficient interfacial electron transfer (TiO_2_ NS/rGO).^51^

Some authors have studied the adsorption process of rGO-modified sponges; however, they did not explore regeneration strategies. In contrast, our work focuses on developing a material that combines the adsorptive properties of the sponge and rGO with the photocatalytic properties of TiO_2_ nanosheets. Few studies in the literature report the use of photocatalysis as a regeneration technology for adsorbents, mainly TiO_2_/activated carbon systems.^30,44,46^

Regeneration of saturated active sites plays a key role in extending the lifetime of such materials. The combination of TiO_2_ nanosheets with UVB irradiation enabled simultaneous removal of OCs via adsorption and photocatalytic degradation. Unlike MS-rGO, recovery methods such as compression^55^ washing with nonpolar solvents, or centrifugation^56^ are ineffective for MS-rGO-4h-TiO_2_ NS when removing OCs. This is likely related to the chemisorption mechanism of adsorption. MB, RhB, IBP, BPA, and TC are aromatic compounds that interact with MS-rGO-4h-TiO_2_ NS primarily through π–π interactions between the OCs and the sp^2^ carbon domains of rGO, or via residual oxygen-containing functional groups (hydroxyl, carboxyl, or epoxy).^55^ The recyclability tests of MS-rGO-4h-TiO_2_ NS (Figure 5h) demonstrated high OC adsorption efficiency, effective regeneration of active sites via photodegradation, and stability of TiO_2_ NS in the material without significant leaching during application. The material could be reused for up to nine cycles without notable loss in removal efficiency for RhB (97%), IBP (71%), and TC (70%). Superhydrophobic/superoleophilic sponges decorated with Zn-based MOF structures^57^ exhibited efficient oil–water separation and antibacterial properties, with 98% removal efficiency maintained over 10 cycles. A dopamine-based biomimetic method produced a superhydrophobic sponge^56^ with 92.1% retention of initial absorption capacity after 35 reuse cycles via centrifugation. A one-step biomimetic approach yielded 3D superhydrophobic/superoleophilic melamine sponges,^52^ retaining 94.32% and 97.76% of initial absorption capacities for diesel and chloroform, respectively, after 20 compression-washing cycles. A 3D porous GO-based sponge was developed for human motion detection.^58^ A melamine/rGO sponge^55^ achieved ∼95% adsorption efficiency for MB solution within 30 s, reaching equilibrium in 90 min with a maximum adsorption capacity of 260.9 mg·g^–1^ by optimizing GO loading and pH.

The enhanced photocatalytic performance with TiO_2_ incorporation into the system MS-rGO, including its adsorption capacity in the absence of irradiation, can be primarily related to oxygen vacancies (OVs). These defects serve as electron donors and acceptors, introducing intermediate energy levels within the bandgap that facilitate electron transitions not observed in defect-free TiO_2_.^59^ By reducing the bandgap energy, OVs enhance photon absorption and promote the separation of photogenerated electron–hole pairs, thereby minimizing recombination and improving photocatalytic efficiency. Additionally, OVs act as active sites for the adsorption of molecules such as O_2_ and H_2_O, enabling redox reactions on the surface.^60^ The integration of rGO and TiO_2_ into MS systems amplified these properties through synergistic interactions. rGO, with its relevant electrical conductivity and large surface area, behaves as an electron acceptor, stabilizing photogenerated charge carriers via an electron transfer state.^61^ Although quantifying OVs is beyond the scope of this study, this synergy is supported by the uniform distribution of TiO_2_ nanoparticles on rGO nanosheets, as confirmed by TEM. The highly defective nature of MS-rGO nanostructures, evidenced by the blue shift and broadening of the G-band in Raman spectroscopy, indicates the presence of both oxygen vacancies and structural defects in rGO, which collectively enhance chemical reactivity. Broad bands in TiO_2_-NS is a strong indicator of oxygen vacancies, particularly when accompanied by a reduced peak intensity, as they reflect lattice disorder and structural defects.

In summary, the MS-rGO-4h-TiO_2_ NS developed in this work demonstrates a unique combination of high adsorption capacity, rapid and uniform GO reduction via microwave-assisted thermochemical synthesis, and efficient photocatalytic regeneration of active sites. Compared to previously reported sponges, our material shows superior performance in crude oil absorption, long-term recyclability, and removal of various emerging contaminants from water. The synergistic interaction between rGO and TiO_2_ nanosheets enhances adsorption, charge separation, and interfacial electron transfer, resulting in effective degradation of organic compounds. These results highlight the potential of MS-rGO-4h-TiO_2_ NS as a versatile and sustainable material for oil–water separation and treatment of contaminated water, offering advantages over conventional PU or GO-based sponges and paving the way for practical applications in environmental remediation. Recent reports demonstrate that hybrid sponges combining graphene derivatives with metal oxides achieve superior oil adsorption efficiencies and mechanical stability, while also enabling additional functionalities such as photocatalytic degradation of organic pollutants under UV irradiation. These multifunctional systems address key challenges related to long-term stability, recyclability, and environmental applicability, reinforcing the relevance of rGO- and TiO_2_-modified melamine sponges for advanced water remediation applications.^62–64^

Thus, the ability of TiO_2_-rGO composites to stabilize charge carriers and enhance photon absorption makes them promising candidates for scalable photocatalytic systems such as MS-rGO-4h-TiO_2_ NS for oil removing from water. Future studies could focus on optimizing the density and distribution of OVs.

## 4 Conclusions

We developed sponges modified with graphene oxide and titanium oxide, whose excellent properties enable their remarkable use in the remediation of water bodies contaminated with various organic pollutants. Their application potential was evaluated under two water contamination scenarios. In the oil contamination scenario, absorption results using MS-rGO-4h reached 91 w/w, while MS-rGO-TiO_2_-4h reached 88 w/w; the presence of TiO_2_ provides greater stability across sponge recovery cycles. In the scenario of water contamination with other organic compounds, such as methylene blue and rhodamine B, adsorption by MS-rGO-TiO_2_ reached up to 99%. TiO_2_ is essential for sponge recyclability, and under UVB lamp irradiation, the compounds could be degraded, allowing the sponge to be reused in a continuous cycle, achieving up to 95% photodegradation of the compound.

These results highlight a synergistic interaction among GO, TiO_2_, and MS, facilitated by a straightforward synthesis method that enables the formation of a hybrid rGO-TiO_2_ structure within the pores of MS. Oxygen vacancies (OVs) play a pivotal role in enhancing the photocatalytic performance, as they introduce defect states that improve charge carrier separation and photon absorption, as supported by Raman spectroscopy and TEM analyses. The simplicity of the methodology presented here offers significant advantages for scalable production, making this hybrid material a promising candidate for large-scale applications in the photocatalytic remediation of water contamination, particularly for the degradation of organic pollutants. Thus, the ability of TiO_2_-rGO composites to stabilize charge carriers and enhance photon absorption makes them promising candidates for scalable photocatalytic systems such as MS-rGO-4h-TiO_2_ NS for oil removing from water. Our findings pave the way for effective water treatment strategies.

## Supplementary Material



## Notes

Lateral size distribution of GO nanosheets obtained from SEM image analysis (PDF) (PDF)
