# Supplementary material for: 3D rGO and rGO/TiO2–Modified Melamine Sponges: Sorbents for Oil and Emerging Organic Pollutant Removal
Source: ACS Omega. 2026 Jan 23;11(5):8580–93. doi: 10.1021/acsomega.5c11602 (PMC12903164; doi:10.1021/acsomega.5c11602)
Supplement: Supplementary file 1 [file ao5c11602_si_001.pdf]

Supporting Information

3D rGO and rGO/TiO_2-Modified Melamine Sponges: Sorbents for Oil and Emerging Organic Pollutant Removal

Kelly Leite dos Santos Castro Assis[a], Thayane Almeida de Medeiros[a], Druval Santos de Sá[a], Carolina Carvalho de Mello[a], Nádia Cristina Da Silva Iack[a], Adriana Maria da Silva[a], Renata Antoun Simão[b], Braulio Soares Archanjo[a] and Carlos Alberto Achete[a]*

[a]Materials Division, National Institute of Metrology, Quality and Technology (Inmetro), Duque de Caxias, Rio de Janeiro (RJ) 25250-020, Brazil.

[b]Department of Materials Science and Engineering, Federal University of Rio de Janeiro (UFRJ),Rio de Janeiro, Rio de Janeiro (RJ) 21941-909, Brazil.

*Corresponding author: klcastro@inmetro.gov.br

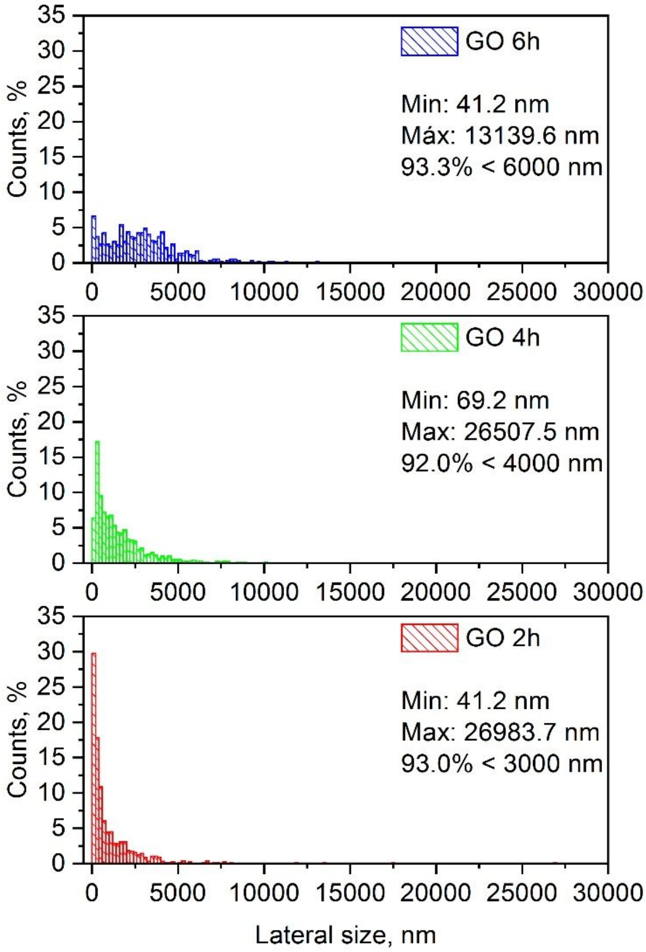

Figure S1. Lateral size distribution of GO nanosheets obtained after different reaction times (2 h, 4 h, and 6 h). The histograms show the frequency of nanosheet sizes measured by SEM image analysis, evidencing a right-skewed distribution typical of exfoliated materials. Increasing the reaction time to 6 h led to the formation of smaller and more uniform nanosheets, while shorter times (2 h and 4 h) resulted in broader and less homogeneous size distributions.

Table S1. Comparative overview of reported sorbent materials used for oil and organic solvent removal from water, highlighting the synthesis process, absorbed materials, absorption capacity (w/w), and sorption cycling performance.

**Material used**	**Synthesis process**	**Absorbed material**	**Absorption capacity (w/w)**	**Cycles Sorption**	**Ref**
Ag/rGO-MS	Chemical reduction & dip-coating	Oils, DCM, Diesel	26–49.2 times	10	[6]
HPCS (MIL-53/PDMS/PU)	Dip-coating and drying	Petrol, Crude oil, Toluene	12–50.5 g/g	6–20	[7]
Ment:Th@Fe3O4-MS	Impregnation with DES and Fe3O4	Various crude oils	101.7–127.3 g/g	85	[8]
DTMS-TiO2@PVF	Cross-linking foaming & silanization	Diesel, Chloroform, MB dye	4.13–10.05 g/g	10	[10]
PP Sponge	Thermally-induced phase separation (TIPS)	Toluene, Chloroform, Fuel oils	5–20 times	Multiple	[11]
MS@Co-ZIF-L	In-situ aqueous growth	n-hexane, Dichloromethane	Efficiency > 99.2%	60	[12]
SGMS (Silk/GO/MS)	Impregnation (Silk as binder)	Chloroform, Silicone oil	30–76.5 g/g	50	[13]
Silanized MF	One-step silanization (MTMS/TEOS)	Crude oil, Chloroform, Diesel	77–163 times	5000	[14]

GMF (Thermal GO/MF)	Thermal reduction (180 °C)	Chloroform, Toluene, Diesel	60–140 times	50	[29]
UMRGMF (Microwave)	Ultrasonic-microwave synergistic method	Chloroform, Diesel, Toluene	Up to 112 times	20	[31]
rGO@MF (Hydrothermal)	Hydrothermal treatment (Hydrazine)	Engine oil, Pump oil, Toluene	90–120 g/g	10	[44]
rGO-MS (Thiourea)	Dip-coating & thiourea reduction	Petrol, Diesel, Chloroform	79–149 g/g	20	[47]
rGOMS (Ascorbic)	Repeated extrusion & AA reduction	n-hexane, CCl4, Pump oil	54.6–132.9 g/g	10	[49]
Hydrochar	Hydrothermal carbonization (HTC)	Methylene Blue (MB dye)	140.35 mg/g	Not specified	[10]
MS-GO (Direct)	Squeezing-sucking method	Methylene Blue (MB dye)	260.9 mg/g	5	[54]
